# A Simple Method to Detect the Inhibition of Transcription Factor-DNA Binding Due to Protein–Protein Interactions In Vivo

**DOI:** 10.3390/genes10090684

**Published:** 2019-09-06

**Authors:** Guangzhe Yang, Dong Chao, Zhenhua Ming, Jixing Xia

**Affiliations:** State Key Laboratory of Conservation and Utilization of Subtropical Agro-bioresources, College of Life Science and Technology, Guangxi University, Nanning 530005, China (G.Y.) (D.C.) (Z.M.)

**Keywords:** protein–DNA binding, protein–protein interactions, yeast one-hybrid, EMSA, ChIP

## Abstract

Binding of transcription factors (TFs) to *cis*-regulatory elements (DNA) could modulate the expression of downstream genes, while interactions between TFs and other proteins might inhibit them binding to DNA. Nowadays, electrophoretic mobility shift assay (EMSA) and chromatin immunoprecipitation (ChIP) approaches are usually employed to detect the inhibitory effect. However, EMSA might not reflect the inhibitory effect in vivo. ChIP requires preparation of specific antibody or stable genetic transformation and complicated experimental steps, making it laborious and time-consuming. Here, based on the yeast one-hybrid (Y1H) system, we present a simple method to detect the inhibition of TF–DNA binding due to protein–protein interactions in vivo. When interactions between TFs and other proteins inhibit TFs binding to DNA, the reporter (Aureobasidin A resistance) gene is not activated, thereby inhibiting yeast growth on media containing the AbA antibiotic. Two examples were tested with the newly developed method to demonstrate its feasibility. In conclusion, this method provides an alternative strategy for detecting the inhibition of DNA-binding of TFs due to their interactions with other proteins in vivo.

## 1. Introduction

Transcriptional regulation is an important way in the regulation of gene expression. Besides RNA polymerases, multiple proteins or transcription factors (TFs) are required to orchestrate the whole transcription event, especially in eukaryotic gene transcription. Transcription is modulated through the binding of TFs to *cis*-regulatory elements usually located upstream of genes. However, the binding of TFs to *cis*-elements may be also regulated by other proteins. Some proteins could interact with TFs, which inhibit them binding to *cis*-elements, thereby affecting the transcription of downstream genes.

Nowadays, EMSA (electrophoretic mobility shift assay) and ChIP (chromatin immunoprecipitation) approaches are usually employed to detect whether interactions between TFs and other proteins inhibited them binding to *cis*-elements [1,2,3,4,5]. For example, the bHLH-type TF PIF1 regulated the DELLA gene *RGA* through binding directly to G-box element (CACGTG) in its promoter [6]. HFR1, an atypical bHLH TF, could interact with PIF1 [2]. In EMSA assay, a clear signal of DNA–protein complex was observed when PIF1 was incubated with a DNA probe containing G-box. However, when HFR1 was co-incubated with PIF1, the intensity of the binding complexes was markedly reduced, and with the increase in HFR1 amount, the binding complexes almost disappeared [2]. So, it was concluded that the HFR1–PIF1 interaction inhibited PIF1 binding to G-box element in vitro [2]. Furthermore, the conclusion was validated with a ChIP assay [2]. Although EMSA assay could visually display the inhibition of the DNA-binding activity of TFs due to their interactions with other proteins, it is conducted in vitro and may not reflect the inhibitory effect in vivo or at the chromatin level. ChIP assay could detect the inhibitory effect at the chromatin level, but it requires preparation of a specific antibody. If universal antibodies are used, proteins of interest with different tags (GFP, FLAG, etc.) should be transformed into target organisms. Additionally, complicated experimental steps are also required in the ChIP assay, which make it time-consuming and difficult to perform. It is therefore desirable to develop a simple method to detect the inhibitory effect in vivo.

Yeast one-hybrid (Y1H) and yeast two-hybrid (Y2H) systems could detect protein–DNA and protein–protein interactions in vivo, respectively. Based on their principles, here we devised a simple method to detect the inhibition of TF–DNA binding due to protein–protein interactions at the chromatin level. Two published examples derived from EMSA and ChIP assays were verified with the newly developed method to demonstrate its feasibility. Also, its disadvantages and a troubleshooting guide are discussed.

## 2. Materials and Methods

### 2.1. Modification of pGBKT7 Vector

To replace the GAL4 binding domain (GAL4-BD), T7 promoter (*P*_T7_), and c-Myc epitope tag (Myc) with the nuclear localization signal (NLS), the pGBKT7 fragment that did not contain GAL4-BD, *P*_T7_, and Myc was amplified with high-fidelity PCR. The amplifying fragment was digested with *Nde*I and *Eco*RI, and ligated with the synthesizing NLS bearing *Nde*I and *Eco*RI overhang (listed in Table S1). The resulting vector was sequenced for verification and named pGBKT7-NLS. To replace ADH1 promoter (*P*_ADH1_) with CYC1 promoter (*P*_CYC1_), the *P*_CYC1_ was amplified from pBT3-SUC vector (Dualsystems Biotech), and the 5’ terminus of the primers was added restriction enzyme site *Bgl*II. The pGBKT7-NLS fragment that did not contain *P*_ADH1_ was amplified with high-fidelity PCR, and the 5’ terminus of the primers was added restriction enzyme site *Bgl*II. The amplifying fragment was digested with *Bgl*II, dephosphorylated with alkaline phosphatase, and ligated with *P*_CYC1_ digested with *Bgl*II. The resulting vector was sequenced for verification and named pmT7.

### 2.2. Vector Construction

To construct bait-specific pAbAi vector, four copies of G-box element with their flanking nucleotides from the *RGA* promoter (listed in Table S1), and three copies of P1BS element with their flanking nucleotides from the *OsIPS1* promoter (listed in Table S1) were synthesized and ligated into the pAbAi vector (Clontech) digested with *Hin*dIII and *Sal*I. The resulting vectors were named G-box-AbAi and P1BS-AbAi. The coding regions of *PIF1* and *HFR1* were amplified from *Arabidopsis* cDNA with high-fidelity PCR. The amplifying *PIF1* fragment was ligated into the pGADT7 vector (Clontech) digested with *Eco*RI and *Sac*I, and pGAD424 vector (Clontech) digested with *Eco*RI and *Pst*I, respectively. The amplifying *HFR1* fragment was ligated into the pGAD424 vector digested with *Eco*RI and *Bam*HI, and pmT7 vector digested with *Xho*I and *Eco*RI, respectively. The coding regions of *OsSPX4*, *OsPHR2*, and its truncated version (PHR-147) were amplified from rice cDNA with high-fidelity PCR. The amplifying *OsPHR2* fragment was ligated into the pGADT7 vector digested with *Nde*I and *Bam*HI. PHR-147 was ligated into the pGADT7 vector digested with *Nde*I and *Eco*RI, and pGAD424 vector digested with *Eco*RI and *Bam*HI, respectively. The amplifying *OsSPX4* fragment was ligated into the pGAD424 vector digested with *Bam*HI and *Bgl*II, and pmT7 vector digested with *Xho*I and *Bam*HI, respectively. The resulting vectors were sequenced for verification. All primers used above are listed in Table S1.

### 2.3. Yeast Experiments

Y1H was performed using the Matchmaker^®^ One-Hybrid System (Clontech, Mountain View, USA). Briefly, the G-box-AbAi and P1BS-AbAi plasmids were linearized with *Bst*BI, and transformed into Y1HGold yeast strain, respectively. Transformants were grown on medium lacking Ura, and the integration of G-box-AbAi and P1BS-AbAi plasmids into the yeast genome was confirmed by PCR. PIF1-424 and P147-424 plasmids were transformed into the G-box-specific and P1BS-specific reporter strain, respectively. Transformants were grown on medium containing Aureobasidin A (AbA). The concentrations of AbA used depend on the bait sequence cloned into pAbAi, and are shown in the figure legends. To test whether protein–protein interactions inhibit protein (TF)-DNA binding, the plasmid combinations of HFR1-mT7/PIF1-424 and SPX4-mT7/P147-424 were transformed into the G-box-specific and P1BS-specific reporter strain, respectively. The double transformed yeast was grown on DDO medium containing AbA. The inhibitory effect could be assessed based on the yeast growth.

## 3. Results

### 3.1. The Principle of the Newly Developed Method

**Figure 1:**
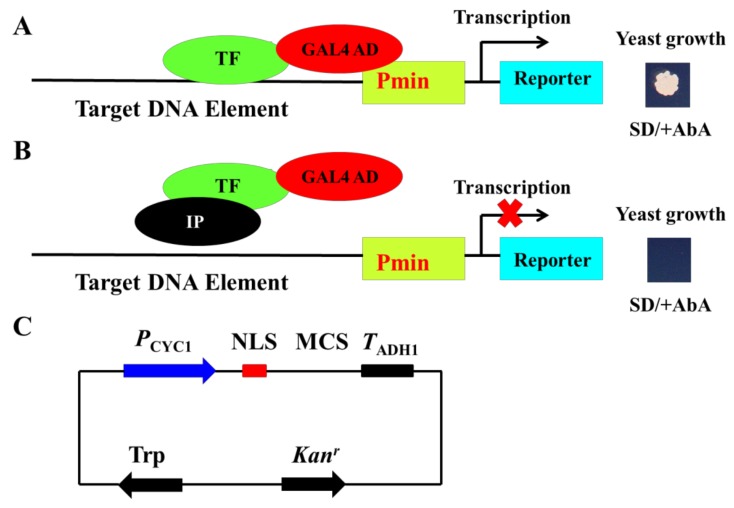
The principle of the newly developed method for detecting the inhibition of transcription factor (TF)-DNA binding due to protein–protein interactions in vivo. (**A**) Schematic diagram of the yeast one-hybrid system. When a TF binds to the target DNA sequence, the GAL4 activation domain (AD) can activate the reporter (AbA resistance) gene expression, which allows yeast to grow on medium supplemented with the AbA antibiotic. (**B**) Schematic diagram of the newly developed method. A TF-interacting protein (IP) is cotransformed with the TF-AD into bait-specific yeast strain. When the interaction between IP and TF inhibits TF binding to target DNA, the reporter gene will not be activated, which inhibits yeast growth on medium containing the AbA antibiotic. (**C**) Vector map of the modified pGBKT7, pmT7. The ADH1 promoter (*P*_ADH1_) and GAL4 DNA-binding domain (DNA-BD) in pGBKT7 are replaced with CYC1 promoter (*P*_CYC1_) and nuclear localization signal (NLS) in pmT7, respectively.

Y1H is used for detecting protein–DNA interactions. The target (bait) DNA is first cloned upstream of a reporter gene, and then transformed into yeast and integrated into its genome or not to generate a bait-specific reporter strain. The TF or target protein is fused to a yeast transcription activation domain (TF-AD), and transformed into the bait-specific reporter strain. If the TF binds the target DNA, the AD activates the expression of reporter gene (Figure 1A). Based on this system, a TF-interacting protein is cotransformed with TF-AD into the bait-specific reporter strain. If interaction between the two proteins inhibits the TF binding to the target DNA, the expression of the reporter gene is not activated (Figure 1B). Thus, based on the observation that the reporter gene is expressed or not, we can determine whether TF–protein interaction inhibits TF binding to its target DNA. This is the principle of our newly developed method.

In the new method, Y1H is performed using Gold Yeast One-Hybrid Library Screening System (Clontech). Multiple studies have demonstrated that the most effective bait-reporter constructs contain at least three tandem copies of the DNA element. The target DNA is cloned into pAbAi vector and integrated into the yeast genome. The reporter is Aureobasidin A (AbA) resistance gene, the activation of which will allow the yeast to grow on the medium containing the AbA antibiotic. The DNA-binding protein (TF) is cloned as downstream fusion to GAL4 AD domain in vectors pGADT7 or pGAD424 (Leu nutritional marker), which differ mainly in the expression level of the fusion protein. The pGBKT7 (Trp nutritional marker) is used to express the TF-interacting protein, and was modified as follows: (1) The GAL4 DNA-binding domain (GAL4-BD) in pGBKT7 is deleted to eliminate its possible interference; (2) GAL4-BD bears nuclear localization signal (NLS), deletion of which might affect the nuclear localization of the TF-interacting protein, so NLS is added at the corresponding position of GAL4-BD. The TF-interacting protein is cloned in the multiple clone site (MCS) downstream of the NLS (Figure 1C); (3) given that high expression of some proteins might be toxic to yeast, the strong promoter ADH1 in pGBKT7 is replaced with a relatively weak promoter CYC1 [7,8] (Figure 1C). The modified pGBKT7 vector is renamed pmT7.

### 3.2. The New Method was used to Verify the Result that HFR1-PIF1 Interaction Inhibited PIF1 Binding to G-box Element

**Figure 2:**
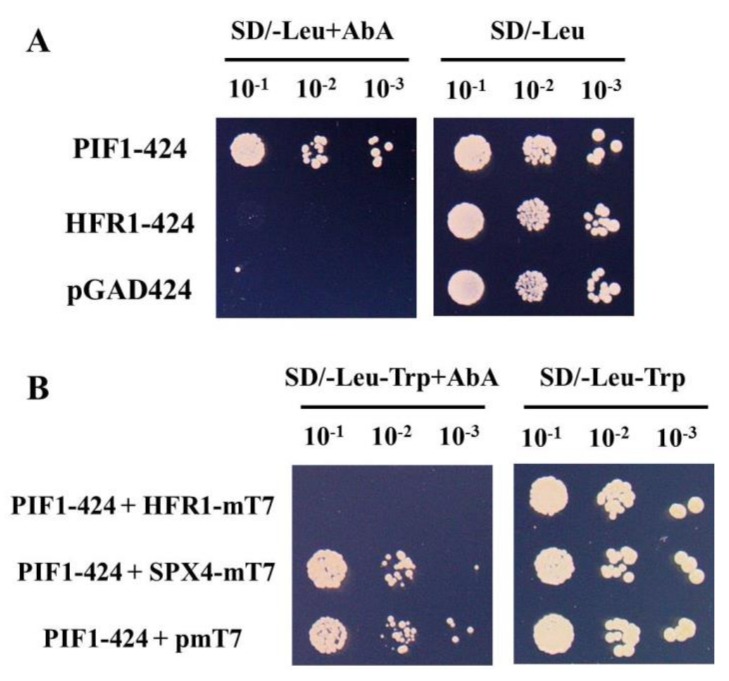
HFR1–PIF1 interaction inhibited PIF1 binding G-box element in yeast. (**A**) Y1H analysis of the binding of PIF1 to G-box element. The PIF1 and HFR1 were cloned into pGAD424 (named PIF1-424 and HFR1-424), respectively, and transformed into reporter strains harboring the AbA resistance gene under the control of a synthetic promoter element consisting of a four repeats of the G-box element from the *RGA* promoter (G-box-specific reporter strain). Dilution series of yeast cells transformed with the indicated plasmids were grown for four days on synthetic complete medium lacking Leu (SD/-Leu, right), and medium lacking Leu but supplemented with 600 ng mL^−1^ AbA (SD/-Leu+AbA, left). (**B**) The new method showing the inhibition of PIF1-G box binding by HFR1–PIF1 interaction. HFR1 and OsSPX4 were cloned into pmT7 (named HFR1-mT7 and SPX4-mT7), and cotransformed with PIF1-424 into G-box-specific reporter strain, respectively. Dilution series of yeast cells transformed with the indicated plasmids were grown for five days on synthetic complete medium lacking Leu and Trp (SD/-Leu-Trp, right) and medium lacking Leu and Trp, but supplemented with 600 ng mL^−1^ AbA (SD/-Leu-Trp+AbA, left).

To test the feasibility of the new method, it was used to confirm the result that HFR1 prevented PIF1 from binding to G-box element in EMSA and ChIP assays [2]. Firstly, four copies of G-box were cloned into the pAbAi reporter vector, and then were integrated into the yeast genome to create a G-box-specific reporter strain. Secondly, PIF1 was cloned into pGADT7 vector (named PIF1-GADT7), and introduced into the G-box-specific reporter strain. However, yeast transformed with PIF1-GADT7 could not grow on medium lacking Leu (SD-Leu, Figure S1), suggesting that the high expression of the PIF1-AD fusion was toxic to yeast cells, so PIF1 was further cloned into the low-level expression vector pGAD424 (named PIF1-424). Meanwhile, PIF1-interacing protein HFR1 was also cloned into pGAD424 (named HFR1-424). Yeast transformed with PIF1-424, HFR1-424, or the empty vector pGAD424 could grow on SD-Leu medium (Figure 2A), indicating that the low expression of the PIF1-AD or HFR1-AD fusion is nontoxic to yeast cells. However, only yeast transformed with PIF1-424, but not HFR1-424 or pGAD424, could grow on medium containing AbA (SD-Leu+AbA, Figure 2A), suggesting that PIF1, but not HFR1, could bind G-box element in yeast, consistent with the observation in EMSA assays [2,6].

Thirdly, HFR1 was cloned into pmT7 vector (named HFR1-mT7), and then cotransformed with PIF1-424 into the G-box-specific reporter strain. Meanwhile, another gene, OsSPX4, that did not interact with PIF1 (Figure S2) was also cloned into pmT7 vector (named SPX4-mT7). The plasmid combinations of SPX4-mT7/PIF1-424 and pmT7/PIF1-424 were also transformed into the G-box-specific reporter strain, respectively, which served as controls for the experiment. Yeast transformed with the plasmid combinations of HFR1-mT7/PIF1-424, SPX4-mT7/PIF1-424, or pmT7/PIF1-424 could grow on the double dropout (DDO) medium (Figure 2B). On DDO medium containing AbA, however, yeast cotransformed with HFR1-mT7 and PIF1-424 could not grow, while the controls, yeast cotransformed with the plasmid combinations of SPX4-mT7/PIF1-424 or pmT7/PIF1-424, could grow (Figure 2B). These results indicated that PIF1 could not efficiently bind the G-box element in yeast cotransformed with HFR1-mT7, while the presence of the empty vector (pmT7) or non-interacting protein OsSPX4 did not interfere with PIF1 binding to the G-box. Furthermore, since HFR1 could not bind the G-box element (Figure 2A), the inhibition of PIF1 binding to G-box element in yeast cotransformed with HFR1-mT7 could not be ascribed to the result that HFR1 competed with PIF1 for binding to the G-box element. In addition, several assays such as Y2H and BiFC (bimolecular fluorescence complementation) have shown that HFR1 directly interacted with PIF1 in vivo (Figure S2) [2]. Taken together, the inhibition of PIF1 binding to G-box element in yeast cotransformed with HFR1-mT7 should originate from the interaction between the two proteins. These results were in agreement with that obtained from EMSA and ChIP assays [2], which demonstrated that the new method could detect the inhibition of TF–DNA binding due to protein–protein interactions in vivo.

### 3.3. The New Method was used to Verify the Result that SPX4–PHR2 Interaction Inhibited PHR2 Binding to P1BS Element

**Figure 3:**
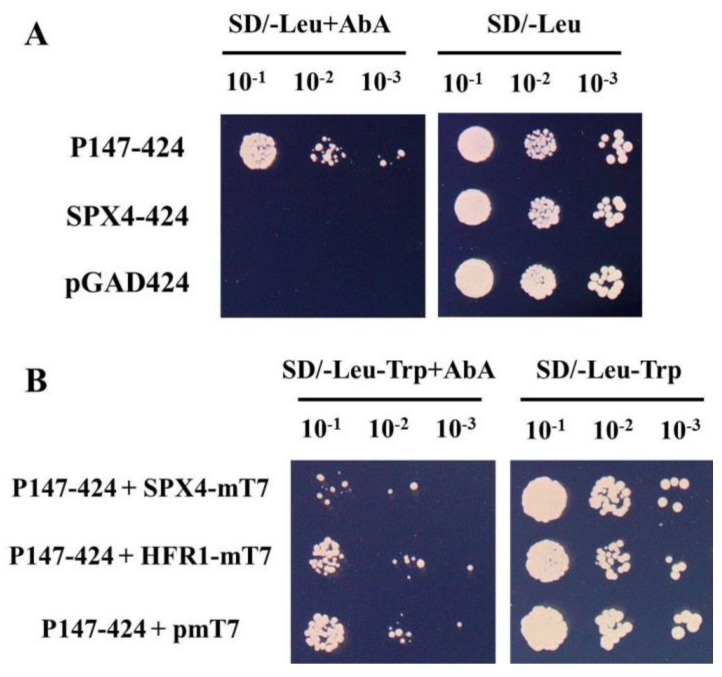
Interaction between SPX4 and OsPHR2-147 (P147) inhibited P147 binding P1BS element in yeast. (**A**) Y1H analysis of the binding of P147 to P1BS element. The P147 and SPX4 were cloned into pGAD424 (named P147-424 and SPX4-424, respectively), and transformed into reporter strains harboring the AbA resistance gene under the control of a synthetic promoter element consisting of a three repeats of the P1BS element from the *OsIPS1* promoter (P1BS-specific reporter strain). Dilution series of yeast cells transformed with the indicated plasmids were grown for 4 days on synthetic complete medium lacking Leu (SD/-Leu, right), and medium lacking Leu but supplemented with 200 ng mL^−1^ AbA (SD/-Leu+AbA, left). (**B**) The new method showing the inhibition of P147-P1BS binding by interaction between SPX4 and P147. SPX4-mT7, HFR1-mT7 and pmT7 were cotransformed with P147-424 into P1BS-specific reporter strain, respectively. Dilution series of yeast cells transformed with the indicated plasmids were grown for 4 days on synthetic complete medium lacking Leu and Trp (SD/-Leu-Trp, right), and medium lacking Leu and Trp but supplemented with 200 ng mL^−1^ AbA (SD/-Leu-Trp+AbA, left).

To confirm the broader utility of the new method, another example was further tested. The plant MYB transcription factor PHRs regulated a series of phosphorus starvation-induced genes through binding to P1BS elements (GNATATNC) in their promoters [9,10]. Using EMSA and ChIP methods, Lv et al. [3] documented that the OsPHR2-interacting protein OsSPX4 could inhibit its binding to P1BS element. The result was further verified with the new method. Firstly, three copies of P1BS elements were cloned into the pAbAi reporter vector, and then integrated into the yeast genome to create a P1BS-specific reporter strain. Secondly, OsPHR2 was cloned into pGADT7 vector (named PHR2-GADT7), and introduced into the P1BS-specific reporter strain. Similarly, yeast transformed with PHR2-GADT7 could not grow on SD-Leu medium (Figure S3), indicating that high expression of the PHR2-AD fusion proteins is toxic to yeast. To eliminate the toxicity of OsPHR2, it was truncated. It has been reported that the C terminus of PHRs containing MYB-CC domains is necessary and sufficient for DNA binding [9], so the C terminus of OsPHR2 containing MYB-CC domains (242-388 aa, PHR-147) was cloned into pGADT7 (named P147-GADT7). Yeast transformed with P147-GADT7 could grow on SD-Leu medium, but it grew slower than that transformed with the empty vector pGADT7, indicating that the high expression of the truncated version of OsPHR2 is still weak toxic to yeast, so it was further cloned into pGAD424 vector (named P147-424). The growth of yeast transformed with P147-424 is similar to that transformed with the empty vector on SD-Leu medium (Figure 3A), suggesting that the low expression of the truncated version of OsPHR2 seemed nontoxic to yeast. On the other hand, yeast transformed with P147-424 could grow on medium containing AbA, while that transformed with the empty vector pGAD424 or SPX4-424 could not grow (Figure 3A), suggesting that the 147-aa fragment of OsPHR2, but not OsSPX4, could bind P1BS elements in yeast. Additionally, Y2H assay showed that truncation of OsPHR2 (PHR-147) did not affect its interaction with OsSPX4, albeit very weak (Figure S4).

Subsequently, SPX4-mT7 was cotransformed with P147-424 into the P1BS-specific reporter strain. Meanwhile, the gene HFR1 (HFR1-mT7) that did not interact with PHR-147 (Figure S4) and pmT7 vector were also cotransformed with P147-424 into the P1BS-specific reporter strain, respectively, which served as controls for the experiment. Similar growth was observed for yeast transformed with different plasmid combinations on the DDO medium (Figure 3B). However, on medium containing AbA, yeast cotransformed with SPX4-mT7 and P147-424 grew markedly slower than that transformed with the plasmid combinations of HFR1-mT7/P147-424 or pmT7/P147-424 (Figure 3B), indicating that PHR-147 could not as efficiently bind the P1BS element in yeast cotransformed with SPX4-mT7 as that in yeast cotransformed with HFR1-mT7 or pmT7. Given the facts that OsSPX4 could not bind the P1BS element in yeast (Figure 3A), and that OsSPX4, but not HFR1, could interact with PHR-147 in yeast (Figure S4), we deemed that the interference of PHR-147 binding to P1BS element in yeast cotransformed with SPX4-mT7 could be attributed to the interaction between the two proteins. These results, again, demonstrated the feasibility of the new method.

## 4. Discussion

The Y1H is a powerful method for detecting DNA–protein interactions, and based on its original system (DNA-centered), multiple modifications have been developed to broaden its applications [11,12,13]. For example, a TF-centered Y1H has recently been developed by Ji et al., which could be used to identify the DNA motifs bound by a defined TF [12]. Also, the Y1H system was modified in the present study, and using the new method, we can determine whether protein–protein interactions inhibit protein–DNA binding by observing yeast growth. If protein–protein interactions inhibit protein–DNA binding, the reporter (AbA resistance) gene is not activated, thereby inhibiting yeast growth on medium with AbA (Figure 1B). Conversely, however, it cannot be determined that protein–protein interactions inhibit protein–DNA binding just by the observation that yeast transformed with the two proteins could not grow on medium containing AbA, as other factors might also inhibit yeast growth, such as competition for DNA binding by the two proteins, or toxicity of the TF-interacting protein to yeast. To rule out these possibilities, rigorous experimental controls must be conducted in the new method. For example, to determine whether the TF-interacting protein could bind to the DNA element as the TF, it must be transformed into the bait (DNA)-specific yeast in parallel with the TF. If yeast transformed with the TF-interacting protein could not grow on medium with AbA (unlike yeast transformed with the TF), it can be determined that the TF-interacting protein is unable to bind the DNA element as the TF, thereby excluding the possibility that the TF-interacting protein competes with the TF for DNA binding. Additionally, to rule out the possibility that the TF-interacting protein is toxic to yeast cells, the yeast transformed with the TF-interacting protein should be spotted onto medium without AbA. If it displays similar growth with that transformed with the empty vector, it can be determined that the TF-interacting protein is nontoxic to yeast. Consequently, it can be determined whether protein–protein interactions inhibit protein–DNA binding in yeast only when all these experimental controls are performed.

The EMSA and ChIP approaches were widely adopted to detect the inhibition of TF–DNA binding due to protein–protein interactions [1,2,3,4,5]. Compared with EMSA, the new method presented here could detect the inhibitory effect in vivo, which might more accurately dissect the transcriptional regulation of genes at the chromatin level. Additionally, the purification of the DNA-binding protein and its interacting protein is not required in the new method, so it does not require high-level expression of the target proteins in heterologous system (for subsequent purification). Consequently, it is especially suited to proteins that are difficult to highly express in heterologous system and purify. Compared with ChIP assay, the new method does not require preparation of specific antibody or the stable genetic transformation of the two proteins (DNA-binding protein and its interacting protein), which is more convenient and less time-consuming.

On the other hand, the method developed in this study is not without disadvantages. The most common problem that we have encountered is that expression of some proteins is toxic to yeast cells. Some solutions to overcome this problem have been provided by the Yeast Protocols Handbook (Clontech). For example, (1) the tested proteins are cloned into low-expression vectors; (2) the tested proteins are truncated, while DNA-binding or protein-interacting activity is still retained. The proteins PIF1 and OsPHR2 used in the present study are highly toxic to yeast cells (Figures S1 and S3). To eliminate the toxicity of PIF1, we replaced the high-expression vector pGADT7 with pGAD424 that allows low expression of the PIF1-AD fusion. As a consequence, yeast with low expression of PIF1 could grow on SD-Leu medium (Figure 2A). To eliminate the toxicity of OsPHR2, it was truncated and cloned into pGAD424. The low expression of the truncated version of OsPHR2 (147 aa) seemed nontoxic to yeast cells and retained the P1BS-binding and OsSPX4-interacting activity (Figure 2A; Figure S4). Toxicity elimination of the tested proteins made the newly developed method feasible and extended its application.

## Supplementary Materials

The following are available online at https://www.mdpi.com/2073-4425/10/9/684/s1, Figure S1: High expression of PIF1 was toxic to yeast, Figure S2: PIF1 could interact with HFR1, but not OsSPX4, in Y2H assay, Figure S3: High expression of OsPHR2 was toxic to yeast, Figure S4: The truncated OsPHR2 (147 aa, P147) could interact with OsSPX4, but not HFR1, in Y2H assay, Table S1: Primers and *cis*-elements used in this study.
