# Supplementary material for: A Simple Method to Detect the Inhibition of Transcription Factor-DNA Binding Due to Protein–Protein Interactions In Vivo
Source: Genes (Basel). 2019 Sep 6;10(9):684. doi: 10.3390/genes10090684 (PMC6770856; doi:10.3390/genes10090684)
Supplement: Supplementary file 1 [file genes-10-00684-s001.pdf]

genes

MDPI

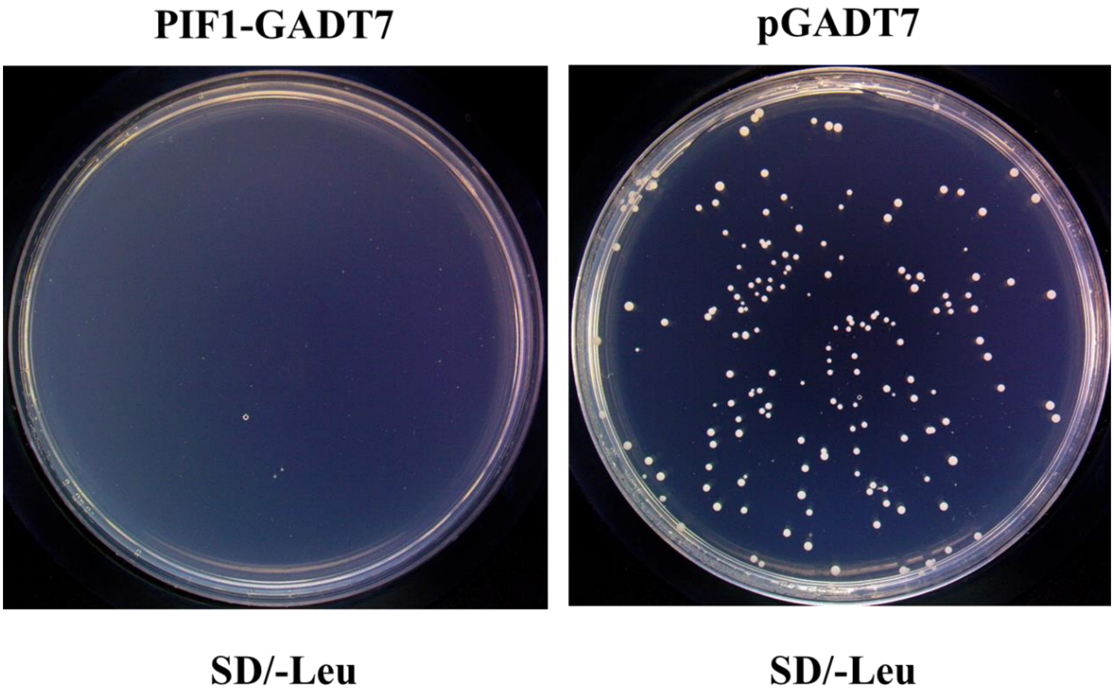

Figure S1. High expression of PIF1 was toxic to yeast. PIF1 was cloned into pGADT7 (named PIF1-GADT7). Yeast transformed with PIF1-GADT7 or empty control vector pGADT7 was grown for 3 d on synthetic complete medium without Leu (SD/-Leu).

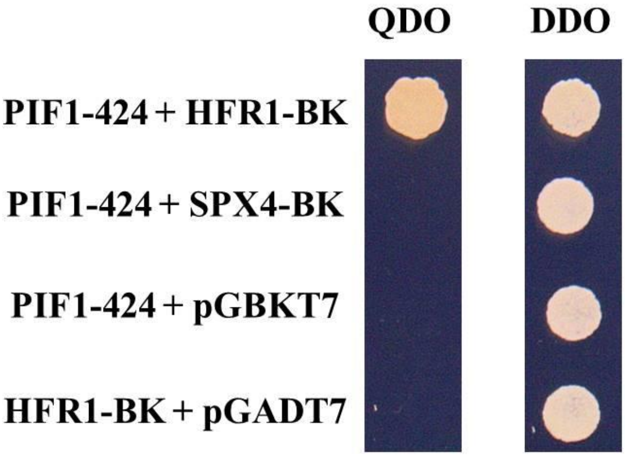

Figure S2. PIF1 could interact with HFR1, but not OsSPX4, in Y2H assay. PIF1 was cloned into pGAD424 (named PIF1-424). HFR1 and OsSPX4 were cloned into pGBKT7 (named HFR1-BK and SPX4-BK), respectively. Yeast transformed with the indicated plasmids were grown for 4 day on synthetic complete medium lacking Leu and Trp (DDO, right), and on medium lacking Leu, Trp, His and Ade (QDO, left).

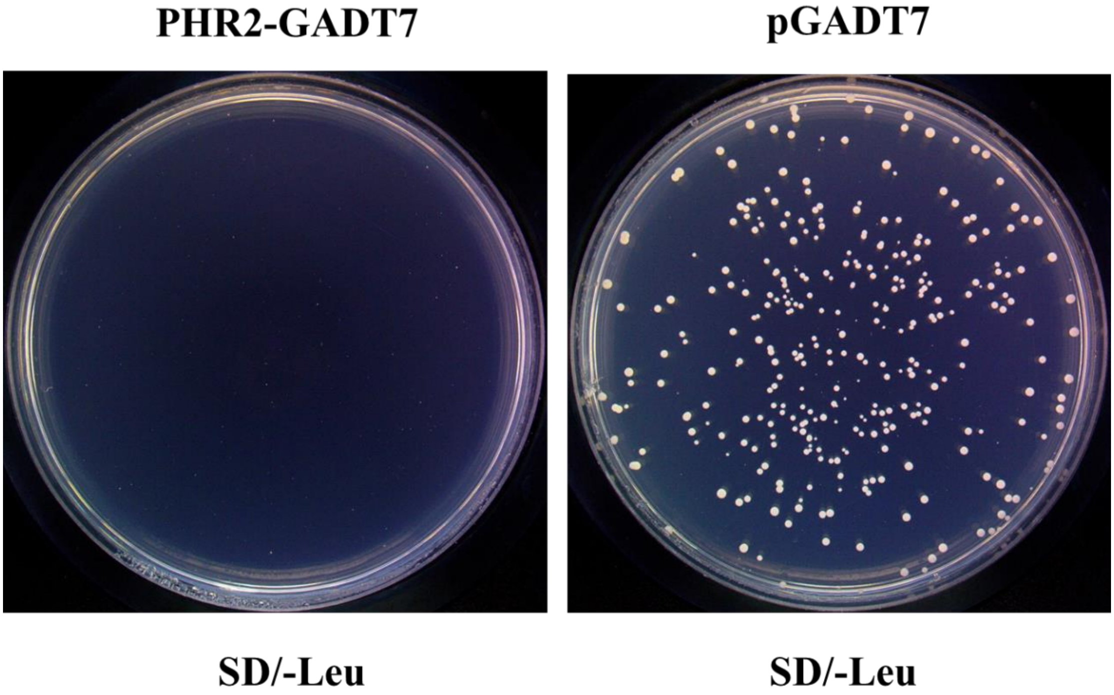

Figure S3. High expression of OsPHR2 was toxic to yeast. OsPHR2 was cloned into pGADT7 (named PHR2-GADT7). Yeast transformed with PHR2-GADT7 or empty control vector pGADT7 was grown for 3 d on synthetic complete medium without Leu (SD/-Leu).

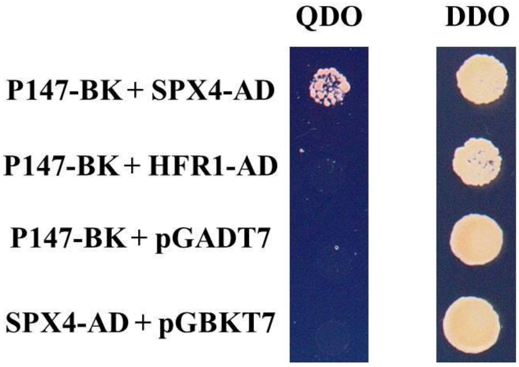

Figure S4. The truncated OsPHR2 (147 aa, P147) could interact with OsSPX4, but not HFR1, in Y2H assay. P147 was cloned into pGBKT7 (named P147-BK). OsSPX4 and HFR1 were cloned into pGADT7 (named SPX4-AD and HFR1-AD), respectively. Yeast transformed with the indicated plasmids were grown for 6 day on synthetic complete medium lacking Leu and Trp (DDO, right), and on medium lacking Leu, Trp, His and Ade (QDO, left).

Table S1. Primers and *cis*-elements used in this study.

Primer Name	Sequence (5′-3′)
**Plasmid Constructs:**	
pGBKT7-NLS	
F	GCCGAATTCCCGGGGATCCGTCGAC
R	ATGGCCATATGCATCTTTCAGGAGGCTTGCTTCAAGC
synthesized NLS	
F	TATGGCGGAATTAATTCCCGAGCCTCCAAAAAAGAAGAGAAAGGTCGAATTGGGTACCGCCCTCGAGGCCATGGAGGCCG
R	AATTCGGCCTCCATGGCCTCGAGGGCGGTACCCAATTCGACCTTTCTCTTCTTTTTTGGAGGCTCGGGAATTAATTCCGCCA
pmT7	
F	GGAAGATCTAAGCTTGAAGCAAGCCTCCTG
R	GGAAGATCTCTGGCGTAATAGCGAAGAGGC
Amplification of P_{CYC1}	
F	GGAAGATCTTCATTTGGCGAGCGTTGGTTG
R	GGAAGATCTTTAGTGTGTGTATTTGTGTTTGCG
PIF1-GADT7	
F	CCGGAATTCATGCATCATTTTGTCCCTGAC
R	GCTCGAGCTCTTAACCTGTTGTGTGGTTTCCG
PIF1-424	
F	CCGGAATTCATGCATCATTTTGTCCCTGAC
R	ATCTCTGCAGTTAACCTGTTGTGTGGTTTCCG
HFR1-424	
F	CCGGAATTCATGTCGAATAATCAAGCTTTCATGG
R	CGCGGATCCTCATAGTCTTCTCATCGCATGG
HFR1-mT7	
F	CCGCTCGAGATGTCGAATAATCAAGCTTTCATGG
R	CCGGAATTCTCATAGTCTTCTCATCGCATGG
PHR2-GADT7	
F	AACCTCATATGATGGAGAGAATAAGCACCAATC
R	CGCGGATCCTTATCTGTCACCTGATTCTG
P147-GADT7	
F	AACCTCATATGACCTCCAACTCCAAGACACG
R	CCGGAATTCTTATGAAGCATCCACCGCCTTG
P147-424	
F	CCGGAATTCACCTCCAACTCCAAGACACG
R	CGCGGATCCTTATGAAGCATCCACCGCCTTG
SPX4-mT7	
F	CCGCTCGAGATGAAATTCGGGAAGGATTTC
R	CGCGGATCCTCATTCATCACGTGGCTGGC
SPX4-424	
F	CGCGGATCCGTATGAAATTCGGGAAGGATTTC
R	GGAAGATCTTCATTCATCACGTGGCTGGC
Y1H	
3×P1BS-F	AGCTTCCTCAGCTCGGATATCCTCAAGATGCCCTCAGCTCGGATATCCTCAAGATGCCCTCAGCTCGGATATCCTCAAGATGCG
3×P1BS-R	TCGACGCATCTTGAGGATATCCGAGCTGAGGGCATCTTGA

	GGATATCCGAGCTGAGGGCATCTTGAGGATATCCGAGCTGAGGA
4×G-box-F	AGCTTGACCCATTAACACGTGGATCCATCACGTGCTG TCAGTTTGAGGACCCATTAACACGTGGATCCATCACGTGCTGTCAGTTTGAG
4×G-box-R	TCGACTCAAACTGACAGCACGTGATGGATCCACGTGTTAATGGGT CCTCAAACTGACAGCACGTGATGGATCCACGTGTTAATGGGTCA

Note: Added restriction enzyme sites are underlined.

© 2019 by the author. Licensee MDPI, Basel, Switzerland. This article is an open access article distributed under the terms and conditions of the Creative Commons Attribution (CC BY) license (http://creativecommons.org/licenses/by/4.0/).
